# A New Acoustic-Based Pronunciation Distance Measure

**DOI:** 10.3389/frai.2020.00039

**Published:** 2020-05-29

**Authors:** Martijn Bartelds, Caitlin Richter, Mark Liberman, Martijn Wieling

**Affiliations:** ^1^Center for Language and Cognition, Faculty of Arts, University of Groningen, Groningen, Netherlands; ^2^Department of Linguistics, University of Pennsylvania, Philadelphia, PA, United States

**Keywords:** acoustic measure, acoustic features, foreign accent, mel-frequency cepstral coefficients, pronunciation, spoken language processing, validation

## Abstract

We present an acoustic distance measure for comparing pronunciations, and apply the measure to assess foreign accent strength in American-English by comparing speech of non-native American-English speakers to a collection of native American-English speakers. An acoustic-only measure is valuable as it does not require the time-consuming and error-prone process of phonetically transcribing speech samples which is necessary for current edit distance-based approaches. We minimize speaker variability in the data set by employing speaker-based cepstral mean and variance normalization, and compute word-based acoustic distances using the dynamic time warping algorithm. Our results indicate a strong correlation of *r* = −0.71 (*p* < 0.0001) between the acoustic distances and human judgments of native-likeness provided by more than 1,100 native American-English raters. Therefore, the convenient acoustic measure performs only slightly lower than the state-of-the-art transcription-based performance of *r* = −0.77. We also report the results of several small experiments which show that the acoustic measure is not only sensitive to segmental differences, but also to intonational differences and durational differences. However, it is not immune to unwanted differences caused by using a different recording device.

## Introduction

The strength of foreign accent in a second language is mainly caused by the first language background of non-native speakers, and is influenced by a wide variety of variables with the most valuable predictor being the age of second-language learning (Asher and Garćıa, 1969; Leather, 1983; Flege, 1988; Arslan and Hansen, 1997). Understanding the factors that affect the degree of foreign accent may be essential for second language teaching, and knowledge about the acoustic features of foreign-accented speech can improve speech recognition models (Arslan and Hansen, 1996; Piske et al., 2001). Computational methods that investigate foreign accent strength are, however, scarce.

Studies that investigate and compare different pronunciations often use transcribed speech (Nerbonne and Heeringa, 1997; Livescu and Glass, 2000; Gooskens and Heeringa, 2004; Heeringa, 2004; Wieling et al., 2011; Chen et al., 2016; Jeszenszky et al., 2017). For example, Kessler (1995) presented the Levenshtein distance for finding linguistic distances between language varieties. To calculate the Levenshtein distance, speech samples have to be manually transcribed using a phonetic alphabet, but this process is very time consuming and labor intensive (Hakkani-Tür et al., 2002; Novotney and Callison-Burch, 2010). Furthermore, transcribing speech is prone to errors, and interference from transcriber variation might lead to a sub-optimal distance calculation when differences in transcribers' habits cannot be distinguished from differences in speakers' productions (Bucholtz, 2007). Another limitation of this approach is that the set of discrete symbols used in phonetic transcriptions is unable to capture all the acoustic details that are relevant for studying accented pronunciations (Cucchiarini, 1996). As Mermelstein (1976) notes, transcribing speech results in a loss of information whereby perceptually distinct differences between sounds diminish or largely disappear. For example, problems may arise when fine-grained pronunciation differences cannot be represented by the set of transcription symbols (Duckworth et al., 1990), or when an important dimension of difference between accents is their use of tone, but no tone or pitch information is transcribed (Heeringa et al., 2009). It is therefore potentially useful to develop an acoustic-only method to study pronunciation differences, such as foreign accent strength in the speech of non-native speakers. Fine-grained characteristics of human speech are preserved in the speech representations, while at the same time a time consuming and costly process may be omitted.

To evaluate computational methods of determining accent differences, validation against reliable data regarding these differences is necessary, which usually consists of comparing the automatically obtained ratings to human judgments of accent strength. Derwing and Munro (2009) stress the importance of including human judgments, since these provide the most appropriate method to evaluate these measurement techniques. Studies that compare human perceptual judgments to a computational difference measure which is not based on the alignment of phonetic transcriptions are uncommon, despite the potential advantages of this approach. This may be due to the challenges of directly comparing speech samples, as there exists a considerable amount of variability in the signal. A substantial amount of variability in the structure of a speech signal is also dependent on non-linguistic characteristics of the speakers, which may mask relevant phonetic information in acoustic measurements (Goslin et al., 2012). For example, Heeringa et al. (2009) calculated speaker-dependent pronunciation distances for a set of fifteen speakers from different Norwegian varieties and for a subset of 11 female speakers. The Manhattan distance was computed between the frequency values of the first three formants per vowel in each word. Correlations between their procedure and human judgments of native-likeness only ranged from *r* = 0.36 to *r* = 0.60 (*p* < 0.001). Given that they only obtained a moderate correlation with the human judgments, their acoustic-based measure could not serve as a reliable alternative to transcription-based methods for assessing accent differences.

The primary goal of this study is therefore to develop an improved acoustic pronunciation distance measure that computes pronunciation distances without requiring phonetic transcriptions. To assess whether the acoustic distance measure is a valid measurement technique to measure accent strength (compared to native speakers), we compare the acoustic distances to a collection of human native-likeness judgments that were collected by Wieling et al. (2014) to evaluate a phonetic transcription-based method. The core of the acoustic distance measure is to use dynamic time warping (DTW) to compare non-native accented American-English to native-accented American-English speech samples represented as Mel-frequency cepstral coefficients (MFCCs). In short, our approach consists of obtaining word-level acoustic differences, which are averaged to obtain speaker-based acoustic differences. To make the comparison less dependent on individual speaker characteristics, we use speaker-based cepstral mean and variance normalization before calculating the word-level acoustic differences. We evaluate the method by comparing the acoustic distances to both transcription-based pronunciation distances and human perception. To better understand what (desired and less desired) differences are captured by our acoustic difference measure, we conduct several small-scale experiments.

## Materials and Methods

### Speech Accent Archive

We use data from the Speech Accent Archive, which contains over 2000 speech samples from both native and non-native American-English speakers (Weinberger, 2015). For each participant an acoustic voice recording of the same standard 69-word-paragraph is present. The paragraph is primarily composed of common English words, and contains a wide variety of consonants and vowels that can be found in the English language. The paragraph is shown in (1).

(1) *Please call Stella. Ask her to bring these things with her from the store: Six spoons of fresh snow peas, five thick slabs of blue cheese, and maybe a snack for her brother Bob. We also need a small plastic snake and a big toy frog for the kids. She can scoop these things into three red bags, and we will go meet her Wednesday at the train station*.

The availability of data from both native and non-native speakers of American-English enables us to compare the accents of a broad range of different speakers of English (Weinberger and Kunath, 2011). Speech samples from 280 non-native American-English speakers make up our target non-native speaker data set, and 115 speech samples from U.S.-born L1 speakers of English serve as our reference native speaker data set. For each non-native speaker the goal is to determine how different that speaker's pronunciation is on average from the native American-English speakers in the reference native speaker data set. We do not rely on choosing a single native American-English reference speaker, as there is considerable regional variability in the data set. The native American-English speakers who rated the non-native speech samples also had different regional backgrounds.

The data we include in this study is similar to the data used for evaluating a transcription-based measurement in the study of Wieling et al. (2014). As in some cases a word was produced twice by a speaker, or two words were merged into one word, we removed duplicate words from the speech samples by deleting one of the repeated words, and merged words were split such that each speech sample consisted of 69 separate words.

Our data set contains slightly more male speakers (206) than female speakers (189). The average age of all speakers in our data set is 32.6 years with a standard deviation of 13.5 years. In the target non-native speaker data set, the average age of starting to learn English is 10.5 years with a standard deviation of 6.6 years. The 280 non-native English speakers have a total of 99 different native languages. The most frequent native languages in the target data set of non-native English speakers are Spanish (*N* = 17), French (*N* = 13), and Arabic (*N* = 12). A total of 46 languages is only spoken by a single speaker.

### Human Judgments of Native-Likeness

Perceptual data have been widely used to assess the degree of foreign-accentedness (Koster and Koet, 1993; Munro, 1995; Magen, 1998; Munro and Derwing, 2001). We therefore use human judgments of native-likeness that were collected in the study of Wieling et al. (2014). They created an online questionnaire in which native speakers of American-English were asked to rate the accent strength of 50 speech samples extracted from the Speech Accent Archive. The degree of native-likeness of the speech samples was judged on a 7-point Likert scale. A score of 1 was assigned to a speaker that was perceived as very foreign-sounding, and a score of 7 was assigned to a speaker that was perceived as having native American-English speaking abilities. The speech samples presented to the participants were not duplicated, so each participant rated each sample at most once. The set of samples available for different participants to judge was changed several times during the period the questionnaire was online. To increase the reliability of the ratings, not all speech samples from the Speech Accent Archive were included in the questionnaire, so that each speech sample could be judged by multiple participants. It was also not compulsory to rate all 50 samples, because the participants could decide to rate a subset of the speech samples.

The questionnaire of Wieling et al. (2014) was distributed by asking colleagues and friends to forward it to native speakers of American-English. The questionnaire was also mentioned in a blog post of Mark Liberman^1^ which led to a considerable amount of responses. In total, 1,143 participants provided native-likeness ratings (57.6% men and 42.4% woman). On average, they rated 41 samples with a standard deviation of 14 samples. The participants had a mean age of 36.2 years with a standard deviation of 13.9 years, and people most frequently came from California (13.2%), New York (10.1%), and Massachusetts (5.9%).

### Experimental Setup

#### Segmentation

We obtain acoustic distances comparing speakers from the target data set to the speakers in the reference data set. The data sets we use contain recordings of the entire 69 word paragraph (henceforth referred to as the complete speech sample). These complete speech samples do not only contain the 69 word pronunciations, but also speech disfluencies. Examples of these disfluencies include, but are not limited to, (filled) pauses, false starts, word order changes, or mispronunciations.

To only compare corresponding segments of speech, we segment each complete speech sample into words. While this segmentation procedure may be performed manually, this is very time consuming (Goldman, 2011). We therefore employ the Penn Phonetics Lab Forced Aligner (P2FA) to time-align the speech samples with a word-level orthographic transcription (Yuan and Liberman, 2008). The P2FA is an automatic phonetic alignment toolkit that is based on the Hidden Markov Toolkit (HTK). Prior to creating the forced alignments, we resample each of the speech samples to 11,025 Hz (Yuan and Liberman, 2008). The forced alignment approach identifies the word boundaries in the speech samples, and by using this information we automatically divide the complete speech samples of the target and reference data set into separate words. Each word corresponds to a word from the elicitation paragraph presented in (1). In this way, we also remove non-speech elements that exist between these word boundaries, preventing them from entering the acoustic distance calculation. After the forced alignment procedure, we have a target data set that for each of the 280 speakers contains 69 segmented speech samples, as well as a reference data set of 115 speakers with for each speaker 69 corresponding segmented speech samples. A detailed explanation of the theoretical framework behind the forced alignment procedure is provided in the studies of Young and Young (1993) and Bailey (2016).

#### Feature Representation

For each segmented speech sample in both data sets, we calculate a numerical feature representation based on Mel-frequency cepstral coefficients (MFCCs). MFCCs have shown their robustness, as these speech features are widely used as representations of phonetic content in automatic speech recognition systems (Davis and Mermelstein, 1980).

**Figure 1:**
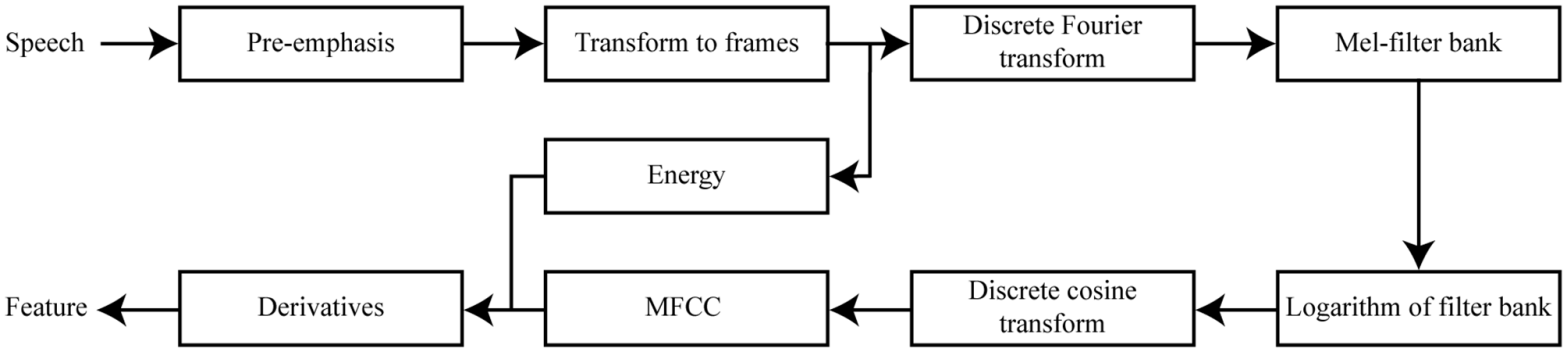
Diagram visualizing the features used in our acoustic distance algorithm.

We visualize the computation of each MFCC feature representation in Figure 1. The first, commonly executed, step in calculating this numerical feature representation is to compensate for the negative spectral slope of each speech sample (Sluijter and Van Heuven, 1996). The nature of the glottal pulses causes voiced segments in the audio signal to contain more energy at the lower frequencies compared to the higher frequencies (Vergin and O'Shaughnessy, 1995). We remove some of these glottal effects from the spectrum of the vocal tract by applying a filter to the audio signal (see Equation 1). This filter emphasizes the higher frequencies, and as a result a more balanced spectrum of the speech sample is obtained. This is usually referred to as the pre-emphasis step (Muda et al., 2010).

(1) $$\begin{array}{r} H\left(z\right)=\text{1 - 0.97}*z^{\text{-1}} \end{array}$$

We then divide each speech sample into short frames of time using a windowing function. These frames of analysis are important since the characteristics of an audio signal are fairly stable when a short frame of time is taken into account (Zhu and Alwan, 2000). We create overlapping frames of a 25 ms time interval using a 10 ms step size. A set of cepstral coefficients is computed for each of these windowed frames per speech sample. The Hamming windowing function is used to extract each frame from the audio signal (Deller et al., 1993).

The Discrete Fourier Transform (DFT) is then taken from each of these windowed frames to transform the audio signal from the time domain to the frequency domain (Zheng et al., 2001). Taking the DFT of the windowed frames is related to the way sound is perceived by human beings. The oscillation of the human cochlea depends on the frequency of incoming sounds, and these oscillations inform the human brain that certain frequencies are present in the audio signal. With the application of DFT, the process that occurs within the human auditory system is simulated (Dave, 2013).

After the DFT is taken from the windowed frames, the Mel spectrum is computed. The DFT-transformed audio signal is modified by passing it through a collection of triangular band-pass filters. These filters are also known as the Mel filter bank, and each processes frequencies that occur within a certain range while discarding frequencies that are outside that range (Muda et al., 2010). The Mel filter bank then provides information about the amount of energy that is present near certain frequency regions (Rao and Manjunath, 2017). The width of the filter banks is determined via Mel-scaling. Units on the Mel scale are based on the way frequencies are perceived by the human auditory system. These Mel units do not correspond to tone frequencies in a linear way, as the human auditory system does not perceive frequencies linearly. Instead, the Mel scale is composed such that the frequencies below 1,000 Hz are approximately linearly spaced, and the frequencies above 1,000 Hz are distributed according to a logarithmic scale (Stevens et al., 1937).

The first filters of the Mel-filter bank are most strict, since the low frequencies are the most informative in speech perception (Raut and Shah, 2015). The energy of voiced speech is mostly concentrated at the lower frequencies (Seltzer et al., 2004). After the DFT-transformed audio signal goes through the triangular-shaped band-pass filters, the logarithm is taken of the energies that are returned by the Mel-filter bank. This procedure is also in accordance with the human auditory system, since humans do not perceive the loudness of an incoming audio signal linearly. The final result of this procedure is a signal that is represented in the cepstral domain (Oppenheim and Schafer, 2004).

The logarithmically transformed filter bank energy representations do, however, overlap. To provide a solution to the overlapping filter banks, the discrete cosine transform (DCT) is computed from the logarithmically transformed filter bank output. The result of the DCT is a set of cepstral coefficients. Following an established standard, we chose to solely include the first 12 cepstral coefficients and energy in each frame, which characterize the most relevant information of the speech signal (Picone, 1993). In addition, we calculate the first-order and second-order derivatives from each of the cepstral coefficients and energy features (Furui, 1981). We therefore have 12 first-order and 12 second-order derivatives that are associated with the 12 cepstral coefficients, and one first-order and second-order derivative related to the energy feature. These first-order and second-order derivatives, or (double) delta coefficients, model the changes between the frames over time (Muda et al., 2010). A total of 39 coefficients is computed at each 10 ms step per speech sample, to represent the most important phonetic information embedded within each 25 ms windowed frame. The MFCC feature representation per segmented speech sample is obtained by concatenating its corresponding vectors of 39 coefficients computed for each of the windowed frames.

#### Normalization

Ganapathy et al. (2011) and Shafik et al. (2009) showed that the quality of the MFCC feature representation is highly influenced by the presence of noise in the speech samples. To reduce the effect of noise, cepstral mean and variance normalization is applied to the feature representations (Auckenthaler et al., 2000). In addition to the robustness in the presence of noisy input, cepstral mean and variance normalization reduces the word error rate in automatic speech recognition implementations, and improves the generalization across speakers (Haeb-Umbach, 1999; Molau et al., 2003; Tsakalidis and Byrne, 2005). Adank et al. (2004) showed that cepstral mean and variance normalization can be used to highlight the linguistic content of the feature representations.

We implement cepstral mean and variance normalization by applying a linear transformation to the coefficients of the MFCC feature representations (Lu et al., 2009). The MFCC feature representations are standardized per speaker by removing the speaker's mean, and scaling to unit variance. The equation that we use to calculate the cepstral mean and variance normalized feature representations is shown in Equation (2).

(2) $$\begin{array}{r} ĉ\left(i,t\right)=\frac{c\left(i,t\right)-\bar{c}\left(i,t\right)}{\sigma \left(i\right)} \end{array}$$

In this equation, the *i*-th cepstral coefficient at time index *t* is represented by *c*(*i, t*). The mean value of each feature representation, and the corresponding standard deviation are given by $\bar{c}\left(i,t\right)$ and σ(*i*), respectively. In Equations (3) and (4), we show how the mean value and standard deviation are obtained. In these equations, *N* corresponds to the number of windows used in processing the speech sample.

(3) $$\begin{array}{r} \bar{c}\left(i,t\right)=\frac{1}{N}*\overset{N}{\underset{t=1}{\sum }}c\left(i,t\right) \end{array}$$

(4) $$\sigma (i)\;=\sqrt{\frac{1}{N}*\sum_{t=1}^{N}(c(i,t)-\bar{c}(i,t){)}^{2}}$$

#### Dynamic Time Warping

The acoustic word distances are computed using the dynamic time warping (DTW) algorithm. This algorithm compares two MFCC feature representations, and returns their degree of similarity as a distance score (Galbally and Galbally, 2015). DTW has already been widely used in the domain of speech recognition, and is also used for sequence comparison in many other research domains, such as computer vision and protein structure matching (Sakoe et al., 1990; Bahlmann and Burkhardt, 2004; Efrat et al., 2007).

To compare a target pronunciation with a reference pronunciation, the DTW algorithm uses the corresponding target and reference MFCC feature representations. These are shown in Equations (5) and (6).

(5) $$\begin{array}{r} \text{target}=\left(x_{1},x_{2},\ldots ,x_{n}\right) \end{array}$$

(6) $$\begin{array}{r} \text{reference}=\left(y_{1},y_{2},\ldots ,y_{m}\right) \end{array}$$

An m*n cost matrix is created to align the target MFCC feature representation with the reference MFCC feature representation (Muda et al., 2010). This cost matrix is filled with the Euclidean distances between every pair of points (frames) in both the target and reference MFCC feature representations (Danielsson, 1980). For example, element (*i, j*) of the cost matrix contains the distance *d* that is given by Equation (7).

(7) $$\begin{array}{r} d\left({\text{target}}_{i},{\text{reference}}_{j}\right)={\left({\text{target}}_{i}-{\text{reference}}_{j}\right)}^{2} \end{array}$$

The optimal alignment between the MFCC feature representations corresponds to the shortest path through the cost matrix, and is therefore to some extent comparable to the edit distance. The DTW algorithm computes the shortest path using an iterative method that calculates the minimum cumulative distance γ(*i, j*) (Keogh and Pazzani, 2001). The cumulative distance is composed of the distance in the current cell *d*(target_*i*_, reference_*j*_) and the minimum of the cumulative distance found in the adjacent cells (shown in Equation 8).

(8) $$\begin{array}{l} \gamma (i,j)=d\left({\text{target}}_{i},{\text{reference}}_{j}\right)\\ \;+min\left(\gamma \left(i-1,j-1\right),\gamma \left(i-1,j\right),\gamma \left(i,j-1\right)\right) \end{array}$$

After the cumulative distance is computed, it is divided by the length of the target feature representation and the reference feature representation (*n*+*m*). It is important to normalize the computed distances, since the speech samples we work with do not necessarily have the same length. Without normalization applied to DTW, longer alignment paths (from longer recordings) would have higher distances than shorter alignments, because they have more frames to accumulate cost (Giorgino et al., 2009).

The final speaker pronunciation distances are obtained by first calculating the acoustic distance for each of the 69 words pronounced by a non-native speaker of American-English and a single native speaker of American-English in the reference data set. We subsequently average these word-based distances to measure the between-speaker acoustic distance. The difference between the pronunciation of a non-native speaker and native American-English in general, is determined by calculating the between-speaker acoustic distances compared to all 115 native American-English speakers, and subsequently averaging these. We compute these acoustic distances for all foreign-accented speech samples by applying this same procedure to each of the 280 non-native speakers of American-English in the target data set. To evaluate our measure, the correlation between the native-likeness ratings and the acoustic distances is computed. We evaluate the impact of the (size of the) set of reference speakers, by calculating the correlation for successively smaller subsets of reference speakers.

### Understanding the Acoustic Distance Measure

In addition to the main experiment, we perform a variety of other analyses to obtain a more complete understanding of the acoustic details captured by the acoustic distance measure.

First we use a multiple linear regression model to predict the human native-likeness ratings on the basis of our acoustic distance measure, but also using the transcription-based distances reported by Wieling et al. (2014), and the (manually counted) number of mispronunciations a speaker made, as these might be important for native-likeness ratings (Flege, 1981), but are not included in either of the two other measures.

Second, to assess whether our acoustic distance measure adequately captures fine-grained segmental differences, we compute acoustic differences between 10 repetitions of hVd words (e.g., [hɪd]) pronounced by a single speaker. We subsequently correlate these differences with differences based on the first and second formant measured at the mid-point of the vowel of the recordings. We follow Wieling et al. (2012) in Bark-scaling the formant-based distances. We use a total of 12 Dutch monophthongs in the vowel context (a, ɑ, ɛ, e, ø, ɪ, i, ɔ, u, o, ʏ, y). We visualize the differences (both the formant-based distances, and the acoustic-based distances) using multidimensional scaling (Torgerson, 1952).

Third and finally, to assess whether non-segmental variability is also captured by our acoustic method, we compute acoustic distances between four series of recordings (10 repetitions) of the word “living”. The first and second series consisted of a normal pronunciation (“living”), but recorded with two recording devices (the built-in microphone of a laptop, and the built-in microphone of a smartphone), the third series consisted of a pronunciation in which the intonation was changed (“living?”), and the fourth series consisted of a pronunciation in which the relative duration of the syllables was changed (“li_ving”).

## Results

**Figure 2:**
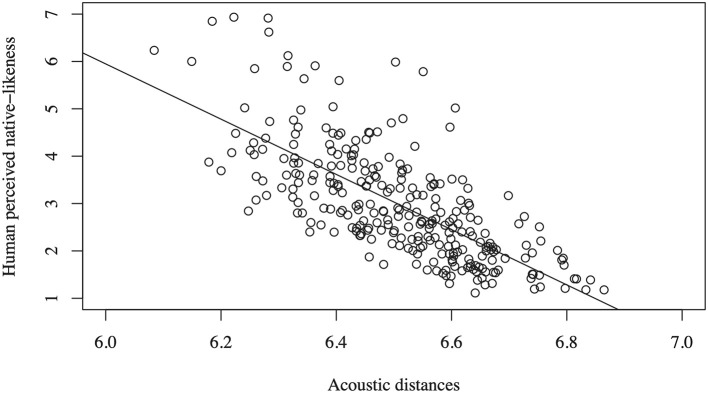
Native-likeness ratings as a function of the computed acoustic distances (*r* = −0.71).

The correlation between the native-likeness ratings and the acoustic distances computed using our acoustic method is *r* = −0.71 (*p* < 0.0001), and therefore accounts for about half of the variance in the native-likeness ratings (*r*^2^ = 0.50). Figure 2 visualizes this correlation in a scatter plot. The acoustic distance measure tends to underestimate the native-likeness (overestimate distances) when the speech samples are rated as being very native-like.

Compared to the transcription-based method of Wieling et al. (2014), who used the Levenshtein distance incorporating automatically determined linguistically-sensible segment distances, and reported a correlation of *r* = −0.77, the performance of our measure is significantly lower (using the modified *z*-statistic of Steiger (1980): *z* = 2.10, *p* < 0.05).

### Impact of Reference Speakers

As the set of reference speakers might affect the correlation, we evaluated the impact of reducing the set of reference speakers. The results are shown in Table 1 and show that the correlation remains comparable, irrespective of the (size of the) reference set (i.e., −0.68 ≤ *r* ≤ −0.72). To assess whether language variation within the set of reference speakers might be important, we computed the acoustic distances using as our reference set (*N* = 14) only the native American-English speakers who originated from the western half of the U.S. and the English-speaking part of Canada. These areas are characterized by less dialect variation compared to the eastern half of the U.S. (Boberg, 2010). Again, this did not substantially affect the correlation, as it remained similar (*r* = −0.70).

**Table 1:** Pearson correlation coefficients *r* between the acoustic distances and human judgments of native-likeness depending on the size of the reference data set.

**Amount of reference speakers**	***r***
10	−0.68
25	−0.71
50	−0.70
75	−0.72

*All correlations are significant at the p < 0.0001 level*.

### Impact of Segmentation and Normalization

Two simplified (baseline) measures, each missing a single component of our acoustic measure, were created to assess how segmentation and cepstral mean and variance normalization of the speech samples contribute to acoustic distances that are more similar to human judgments of native-likeness. The results of this experiment is shown in Table 2. It is clear that not using the normalization approach is much more detrimental than not segmenting, but that the best results are obtained when doing both. The modified *z*-statistic of Steiger (1980) indicates that our acoustic method significantly outperforms either of the two simpler methods (*z* = 4.11, *p* < 0.0001).

**Table 2:** Pearson correlation coefficients *r* of acoustic distances compared to human judgments of native-likeness, using different methods to compute the acoustic distances.

**Model**	***r***
Baseline 1 (only segmentation)	−0.27
Baseline 2 (only normalization)	−0.63
Acoustic measure (segmentation and normalization)	−0.71

*All correlations are significant at the p < 0.0001 level*.

### Understanding the Acoustic Distance Measure

We fitted a multiple linear regression model to determine whether the acoustic distance measure and the transcription-based distance measure captured distinctive aspects of pronunciation. We also assessed the influence of the number of mispronunciations. The coefficients and associated statistics of the predictors used are shown in Table 3. The results show that the transcription-based distances and acoustic distances both contribute significantly to the model fit (*p* < 0.05). This is not the case for the amount of mispronunciations per speaker in the target data set (*p* >0.05). The presented model accounts for 65% of the variation in the human judgments of native-likeness (*r*^2^ = 0.65). Only using the transcription-based distance measure accounted for 60% of the variation. Consequently, our acoustic measure also seems to capture information which is not present in phonetic transcriptions.

**Table 3:** Coefficients of a multiple regression model predicting human judgments of native-likeness.

	**Estimate**	**Std. Error**	***t*-value**	***p*-value**
Intercept	24.19	2.68	9.04	<0.001
Transcription-based distances	−379.30	34.26	−11.07	<0.001
Acoustic-based distances	−2.79	0.44	−6.35	<0.001
Amount of mispronunciations	0.01	0.03	0.26	0.795

The results in Table 4, show that our acoustic measure can capture both intonation and timing differences as these lead to larger distances than comparing individual repetitions of the same word pronounced by the same speaker. However, it also shows that when recording the normal pronunciation by two microphones simultaneously, the acoustic distances between the two simultaneous recordings are higher than zero, whereas the pronunciation is in fact identical. Note that the acoustic distance when comparing the 10 normal pronunciations is also not zero, due to small deviations in the pronunciations.

**Table 4:** Averaged acoustic distances and standard errors of four variants of the word “living”.

	**Compared to normal pronunciation**
Normal pronunciation	4.35 (0.50)
Normal pronunciation (different recording device)	6.94 (0.15)
Rising intonation	7.12 (0.13)
Lengthened first syllable	6.65 (0.13)

**Figure 3:**
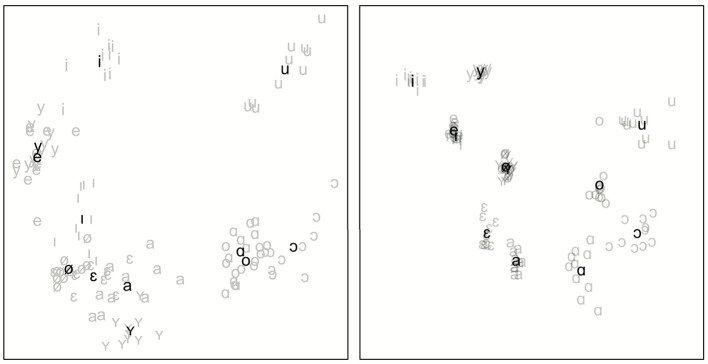
MDS plots visualizing the acoustic vowel distances **(left)** and the formant-based vowel distances **(right)**. Individual pronunciations are shown in light gray, whereas the averages per vowel are shown in black.

Another indication of how well our acoustic measure captures segmental information is shown by the significant positive correlation of *r* = 0.68 (*p* < 0.0001) between the formant-based acoustic vowel differences and the computed acoustic differences between the hVd-words. Figure 3 shows these relative vowel distances by using a multidimensional scaling visualization of the formant-based vowel differences (visualizing all variation) and the DTW-based vowel differences (visualizing 47% of the variation in the original differences).

## Discussion

We have created an acoustic-only approach for calculating pronunciation distances between utterances of the same word by different speakers. We have evaluated the measure by calculating how different the speech of non-native speakers of American-English is from native American-English speakers, and by comparing our computed results to human judgments of native-likeness. While our method is somewhat outperformed (*r* = −0.71 vs. *r* = −0.77) by the transcription-based method introduced by Wieling et al. (2014), our measure does not require phonetic transcriptions, whose production is time consuming and prone to errors. Given that our method is fully automatic, the trade-off in performance may be worthwhile.

Word segmentation and especially speaker-based cepstral mean and variance normalization of the MFCC speech representations were important in creating an adequate acoustic-based distance measure. These results show the importance of pre-processing continuous speech samples, as the comparison of pronunciations in speech samples is most reliable when it is based on comparable and normalized segments of speech that we obtain from word-level forced alignment.

The multiple regression model showed that the acoustic distance measure explained variance not accounted for by the transcription-based distance measure. Particularly, our further experiments showed that our measure is both sensitive to timing and intonation differences. However, the measure is also sensitive to different recording devices, which is undesirable and may partly explain why the method is outperformed by the transcription-based method. While the MFCC feature representation with cepstral mean and variance normalization attempts to minimize non-linguistic confounds, it is only partly successful, as a computational representation of general phonetic information remains a difficult issue in speech processing technology (Gemmeke et al., 2011).

Consequently, future work should investigate whether other acoustic (pre-processing) techniques may improve our acoustic measure. For example, contextual acoustic encoding techniques related to word embeddings like *wav2vec* and *vq-wav2vec* may highlight acoustic details that are linguistically relevant (Baevski et al., 2019; Schneider et al., 2019). Additionally, generating a shared phonetic space through which two speech samples may be compared (Ryant and Liberman, 2016) may be useful. Nevertheless, our work serves as a useful and promising starting point for a fully automatic acoustic pronunciation distance measure.

## Data Availability Statement

The code and datasets generated for this study are available at: https://github.com/Bartelds/acoustic-distance-measure.

## Author Contributions

MB and MW conceptualized the research. MB and CR designed and conducted the experiments, performed data analysis, and data visualization. MB wrote the first version of the manuscript. All authors contributed to manuscript revision, read, and approved the submitted version.

## Conflict of Interest

The authors declare that the research was conducted in the absence of any commercial or financial relationships that could be construed as a potential conflict of interest.

## Footnotes

^1^https://languagelog.ldc.upenn.edu/nll/?p=3967, May 19, 2012, “Rating American English Accents.”
